# Intrinsically Disordered and Aggregation Prone Regions Underlie β-Aggregation in S100 Proteins

**DOI:** 10.1371/journal.pone.0076629

**Published:** 2013-10-01

**Authors:** Sofia B. Carvalho, Hugo M. Botelho, Sónia S. Leal, Isabel Cardoso, Günter Fritz, Cláudio M. Gomes

**Affiliations:** 1 Instituto Tecnologia Química e Biológica, Universidade Nova de Lisboa, Oeiras, Portugal; 2 Molecular Neurobiology Unit, Instituto de Biologia Molecular e Celular, Porto, Portugal; 3 Escola Superior Tecnologia Saúde Porto, Instituto Politécnico, Porto, Vila Nova de Gaia, Portugal; 4 Department of Neuropathology, University of Freiburg, Freiburg, Germany; Consejo Superior de Investigaciones Cientificas, Spain

## Abstract

S100 proteins are small dimeric calcium-binding proteins which control cell cycle, growth and differentiation via interactions with different target proteins. Intrinsic disorder is a hallmark among many signaling proteins and S100 proteins have been proposed to contain disorder-prone regions. Interestingly, some S100 proteins also form amyloids: S100A8/A9 forms fibrils in prostatic inclusions and S100A6 fibrillates *in vitro* and seeds SOD1 aggregation. Here we report a study designed to investigate whether β-aggregation is a feature extensive to more members of S100 family. *In silico* analysis of seven human S100 proteins revealed a direct correlation between aggregation and intrinsic disorder propensity scores, suggesting a relationship between these two independent properties. Averaged position-specific analysis and structural mapping showed that disorder-prone segments are contiguous to aggregation-prone regions and that whereas disorder is prominent on the hinge and target protein-interaction regions, segments with high aggregation propensity are found in ordered regions within the dimer interface. Acidic conditions likely destabilize the seven S100 studied by decreasing the shielding of aggregation-prone regions afforded by the quaternary structure. In agreement with the *in silico* analysis, hydrophobic moieties become accessible as indicated by strong ANS fluorescence. ATR-FTIR spectra support a structural inter-conversion from α-helices to intermolecular β-sheets, and prompt ThT-binding takes place with no noticeable lag phase. Dot blot analysis using amyloid conformational antibodies denotes a high diversity of conformers; subsequent analysis by TEM shows fibrils as dominant species. Altogether, our data suggests that β-aggregation and disorder-propensity are related properties in S100 proteins, and that the onset of aggregation is likely triggered by loss of protective tertiary and quaternary interactions.

## Introduction

The S100 protein family represents the largest subgroup within the Ca^2+^-binding EF-hand protein superfamily. These proteins are only expressed in vertebrates, thus suggesting that they are evolutionary young. In humans, 21 different members involved in a wide variety of both intra and extra-cellular processes have been identified to date [1,2]. Many S100 proteins exhibit cell- and tissue-specific expression patterns as well as specific subcellular localizations, pointing towards a high degree of specialization among them. They play a central role in the regulation of several cellular processes including cell cycle, cell growth, differentiation and motility. Underlining the importance of S100 proteins in signaling, altered expression levels of several S100 proteins are implicated in numerous human disorders. This is the case in many types of cancer [3], neurodegenerative disorders such as Alzheimer’s disease (AD) [4-7], inflammatory and autoimmune diseases [3,8]. Therefore, S100 proteins hold significant interest as potential therapeutic targets. S100 proteins exhibit key structural features common to all members of the family [9]. Each S100 domain is about 10-12 kDa in size and contains two EF-hand helix-loop-helix structural motifs arranged in a back-to-back manner and connected by a flexible hinge [9]. The structure and biological activity of S100 proteins are modulated by metal ions, including calcium, zinc and copper. The binding of Ca^2+^ via EF-hand motifs triggers conformational changes that lead to the exposure of an inter-helical hydrophobic protein interaction site [9]. Some S100 proteins also bind Zn^2+^ and Cu^2+^ at secondary binding sites with high affinity but this is usually associated with subtle conformational changes [10]. These metal binding properties of S100 proteins have an important role in the modulation of their folding, oligomerization state and function as in S100A12 [11,12], S100A8/A9 [13,14] and S100B [15]. A recent assessment suggested a high degree of intrinsic disorder in some regions within S100 proteins, which was hypothesized to correlate with their broad interactome [16], although S100-target interactions occur largely via preformed target interaction sites. In any intrinsically disordered segments are known to be particularly susceptible to misfolding and aggregation [17-20], and it is interesting that amyloid β-aggregates have been identified in some S100 proteins. For example the pro-inflammatory S100A8/A9 heterodimer was found in amyloid deposits from prostate cancer patients, in inclusions called *corpora amylacea*. Subsequent studies showed that this protein forms amyloid oligomerization and fibrillation also *in vitro* [21,22]. Recently, we showed that S100A6 forms amyloid fibrils under physiological conditions and that the native protein nucleates Cu/Zn superoxide dismutase (SOD1) fibrillation, shortening its nucleation process. These findings suggest a novel role for S100A6 aggregation in human neuropathologies, particularly in amyotrophic lateral sclerosis (ALS) [23]. Taking into account this background, we herein report a study in which we have analyzed seven human S100 proteins for relationships between aggregation propensity, disordered regions and amyloid formation, combining computational and biophysical tools.

## Materials and Methods

### Chemicals and Proteins

All reagents were of the highest grade commercially available. Thioflavin T (ThT) and 8-Anilino-1-naphthalenesulfonic acid (ANS) were obtained from Sigma. A Chelex resin (Bio-Rad) was used to remove contaminant trace metals from all solutions. S100 proteins were expressed and purified to homogeneity using previously established protocols for S100A2, S100A3, S100A4, S100A6, S100A12, S100B [24,25] and S100A8/A9 [13]. Protein concentration was determined using Bradford’s method [26].

### Aggregation and disorder propensity analysis

The amyloidogenic propensity of S100 proteins was computed using the Zyggregator [27,28] and Waltz [29]. Multiple sequence alignment was performed in ClustalX2. Intrinsic disorder prediction was performed using the VSL2P predictor [30,31].

### Aggregation Assays

Amyloidogenesis assays were performed as described previously [24], monitoring ThT emission. Briefly, S100 proteins were diluted into 50 mM Glycine, pH 2.5, 5 mM TCEP, 5 mM EDTA and 100 mM NaCl. Final S100 protein concentration was 1 mg/ml and a ratio of ThT: protein of 2 was used. Amyloid formation was promoted by quiescent incubation at 37 °C. Real time ThT fluorescence emission at 480 nm was recorded using a BMG Fluostar Optima fluorescence plate reader upon excitation at 440nm. Curve analysis and lag time determination was carried out as described in [32,33]. The lag time was calculated at different initial time ranges for the different proteins: S100A4 (0-60 h), S100A6 (0-21 h), S100A8/A9 (0-160 h), S100A12 (0-30 h).

### Transmission Electron Microscopy

For visualization by TEM, 5 µl sample aliquots were absorbed to carbon-coated collodion film supported on 400-mesh copper grids, and negatively stained with 1% uranyl acetate. The grids were exhaustively visualized with a Jeol microscope (JEM-1400), operated at 80 kV.

### Dot-blot Analysis

The reactivity of S100 oligomers against conformation-dependent antibodies was carried out in a dot-blot analysis as described in [34] using the anti-fibrillar oligomers and fibrils OC antibody (AB2286 Merck Millipore) and the A11 anti pre-fibrillar oligomers antibody (AB9234 Merck Millipore) at pH 2.5 at time 0h and after 160 h of incubation. α-synuclein aggregates (oligomers and fibrils) and Aβ (fibrils) are included as controls for the reactivity of the OC and A11 conformational antibodies.

### ANS binding assay

ANS fluorescence emission spectra were recorded between 400 nm and 600 nm in a Cary Varian Eclipse instrument using 370 nm as excitation wavelength. Samples of 10µM apo S100 proteins were prepared in 50 mM Glycine, pH 2.5, 5 mM TCEP, 5 mM EDTA and 100 mM NaCl at 25°C and incubated for 30 min. After addition of 100 µM ANS the samples were incubated another 30 min.

### ATR FT-IR

Infrared absorption spectra were used to carry out a fast analysis of protein secondary structure [35] and were acquired on a Bruker IFS 66/S spectrometer equipped with a MCT detector and a thermostatized Harrick BioATR II cell. All measurements were obtained with 8 mg/mL of apo S100A6 at pH 2.5 or 7.0. Samples were never allowed to dry on the measuring surface. Each spectrum represents the mean of 150 scans taken at a resolution of 4 cm^-1^. Band assignments were based on typical absorption regions for specific secondary structure elements [36].

### Analytical size exclusion chromatography

Analytical SEC was performed at room temperature on a Superdex 75 Tricorn high performance column (GE Healthcare, V_column_ =24 ml) connected to an AKTA Purifier UPC-10 system and run at 0.5 ml/min. The column was calibrated with proteins of known molecular weight: Fd. *A. ambivalens* (16 kDa), cytochrome c (11.8 kDa), ribonuclease A (13.7 kDa), monomeric SOD1 (16 kDa), chymotrypsinogen (25 kDa), dimeric SOD1 (32 kDa), ovalbumin (43 kDa) and albumin (67 kDa). 100 µl of S100A6 at 0.5mg/ml was applied and run at pH 3 and 7: running buffers were 50mM Glycine (pH 3) or Tris-HCl (pH 7) with 5mM TCEP, 5mM EDTA and 150mM NaCl. pH was set to 3 instead of 2.5 due to the chemical stability range of the column used.

## Results

### Aggregation propensity and local disorder in S100 proteins

**Figure 1:**
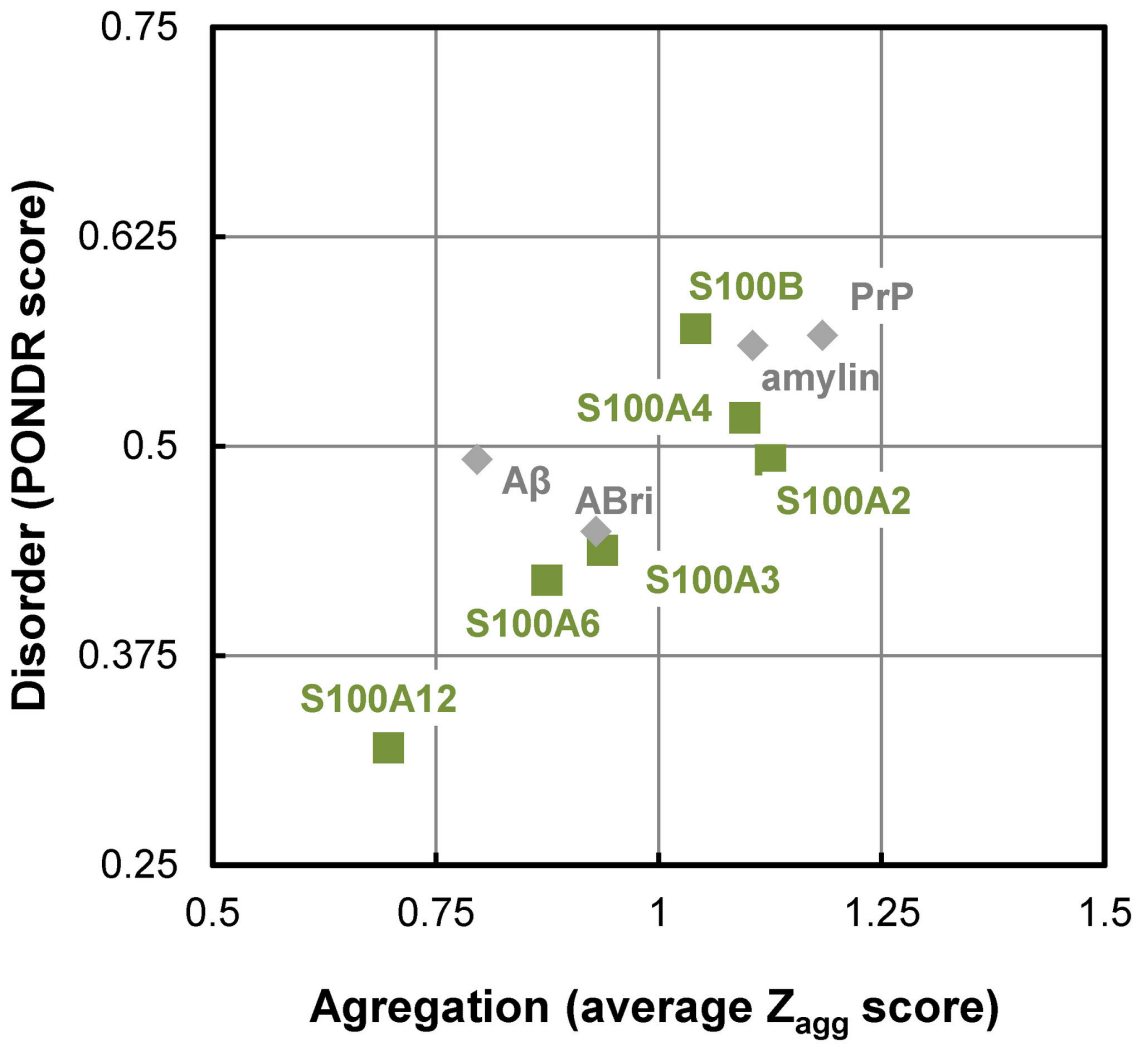
Correlation between aggregation propensity and disorder scores for S100 proteins. Plot of weighted average aggregation propensity (average Z_agg_) versus average disorder (PONDR) scores computed using the Zyggregator and VSL2P algorithms, respectively. Squares stand for to the studied S100 proteins and diamonds for well-known proteins with bona fide disordered regions (Aβ, PrP, amylin and ABri, as compiled in [18]. The threshold for high aggregation propensity is 1 (Z_agg_>1) and for intrinsic disorder is 0.5 (PONDR>0.5).

A set of human S100 proteins representing the diverse functional diversity of this group was selected to investigate if β-aggregation is a property extensive to more members of this protein family (Table 1). The selected S100 proteins are involved in diverse biological functions and represent members of different subgroups within the S100 protein family [1]. Intrinsic disorder is a hallmark of signaling proteins as it affords the structural flexibility required for interactions with multiple partners [37]; very recently this property has been suggested to be inherent to many S100 proteins [16]. Since β-aggregation and amyloid fibril formation are characterized by cross β-structures, it has been proposed that intrinsic disorder in proteins promotes this type of aggregation reactions [18]. In order to investigate the relationship between these two properties in S100 proteins we first analyzed the aggregation propensities using the Zyggregator algorithm [28]. Interestingly, all seven S100 proteins display high global with Z_agg_>0.63; S100A2, S100A4 and S100B even reveal aggregation scores above the threshold for high aggregation propensity (Z_agg_>1). As a next step we compared how the mean aggregation score relates to the disorder score for the studied proteins, as calculated in [16]. A plot of the predicted disorder versus the aggregation score analysis showed a linear correlation (r^2^ = 0.965) between the aggregation and disorder scores indicating that these two properties are related to each other in the analyzed S100 proteins (Figure 1). Interestingly, a number of proteins involved in amyloid diseases which contain disordered segments revealed a similar trend (amylin, prion protein and ABri peptide). Indeed, many amyloid deposits formed in neurodegenerative diseases are caused by precursor proteins with high intrinsic disorder. Nevertheless, β-aggregation is also known to depart from globular states, as a result of conformational fluctuations that favor higher energy conformers which in parts expose aggregation prone regions (reviewed in [28,38]).

**Table 1:** Functions, disease associations and representative structures of the analyzed S100 Proteins.

**Protein**	**Functions**	**Disease association**	**Representative structure (PDB ID)**	**References**
S100A2	Tumor suppressor protein	Cancer	2RGI	[54,55]
S100A3	Highly expressed in some astrocytomas. Role in epithelial cell differentiation and in calcium-dependent hair cuticular barrier formation.	Cancer	1KSO	[56,57]
S100A4	Biological role of nuclear S100A4 is uncharacterized. Angiogenic effects and metastatic progression.	Cancer	1M31	[58-61]
S100A6	Cell proliferation, cytoskeletal dynamics and tumorigenesis	Cancer. Amyotrophic Lateral Sclerosis	1K9P	[1,62]
S100A8/A9	Expressed predominantly in phagocytes. Chemotactic molecule in inflammation.	Inflammatory disorders	1XK4	[1,13]
S100A12	Expressed predominantly in phagocytes. Involved in host-parasite response	Inflammatory disorders	2WCE	[1,11]
S100B	Expressed in astrocytes and in other neuronal populations. Neurotrophic activity. At nanomolar levels promotes neuron survival and growth. At micromolar levels leads to apoptosis.	Neurodegeneration. Alzheimer’s Disease, Down Syndrome, Multiple Sclerosis	2PRU	[63,64]

### Tandem arrangement of disordered and aggregation prone segments in S100 structures

**Figure 2:**
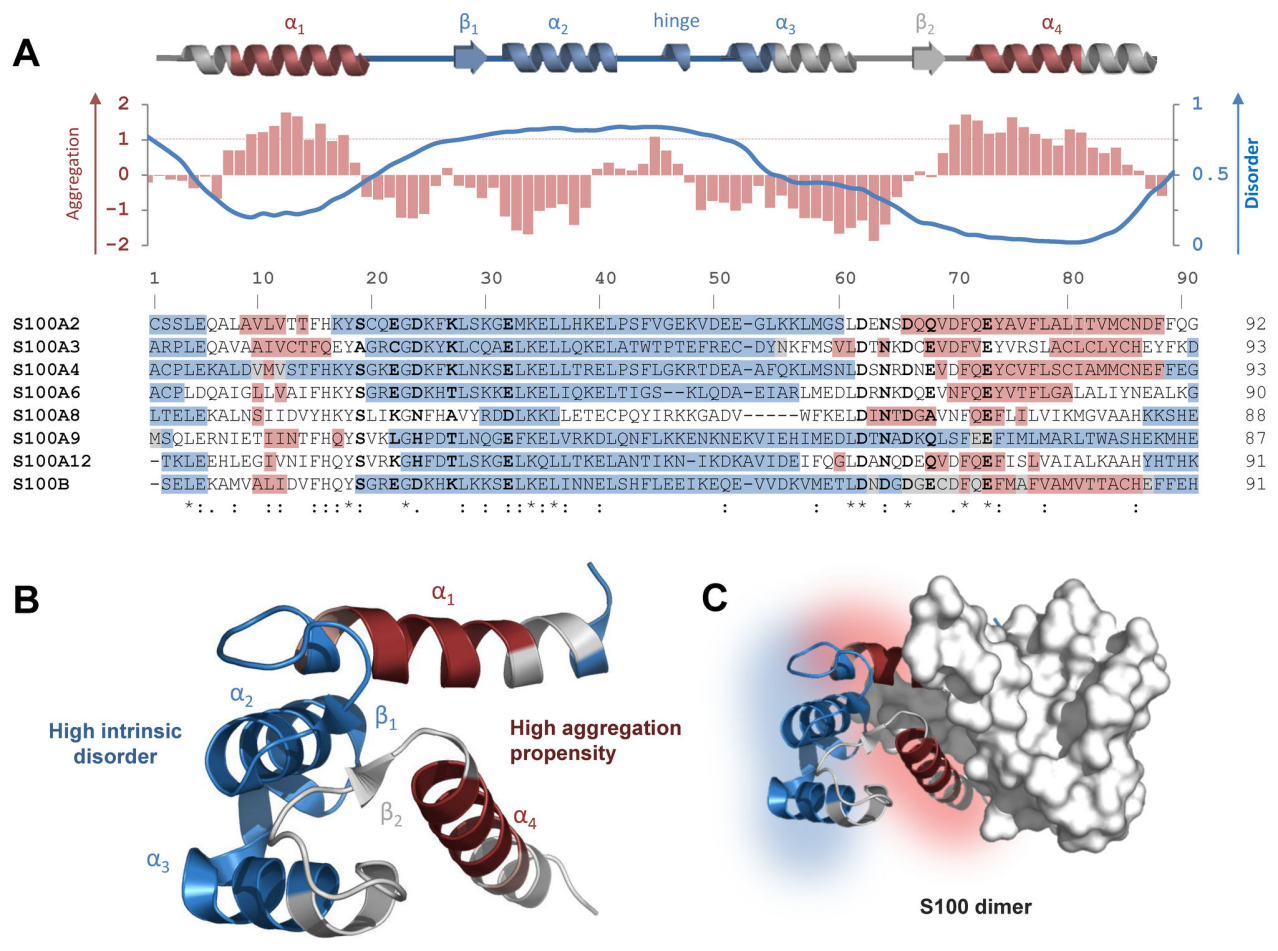
Position specific analysis and structural mapping of tandem disordered and aggregation-prone segments in S100 proteins. (A) The top scheme is a linear representation of the secondary structure elements in S100 proteins. The red bars in the plot stand for average aggregation propensity within S100 proteins. The blue curve was redrawn from [16] and represents the average disorder PONDR VSL2P score for S100 proteins. The threshold for high aggregation propensity is 1 (Z_agg_>1) and for intrinsic disorder is 0.5 (P>0.5). Below is a multiple sequence alignment of the studied S100 proteins. The residues with values above the thresholds for aggregation and intrinsic disorder scores for individual proteins are highlighted in red and blue, respectively. The residues with values above both thresholds are highlighted in gray. The Ca^2+^-coordinating residues in the S100 and canonical EF-hands are highlighted in bold. (B) Representation of high aggregation propensity regions (red) of S100 proteins located in helices α1 and α4 (Z_agg_>1), which are contiguous to locally disorder-prone segments (blue) mainly in helices α2 and α3 and on the hinge region (PONDR>0.5). (C) Representation of a S100 dimer highlighting the segments with high propensity within helices α1 and α4 located at the dimer interface, to illustrate the structural protection afforded by dimer assembly.

In order to further explore this relationship we have proceeded with a position specific analysis and structural mapping of the disorder and aggregation propensities in the analyzed S100 proteins. Aggregation and disorder scores were independently computed for individual sequences and the analyzed S100 proteins displayed a very similar distribution of aggregation and disorder -prone regions. Since S100 proteins have very similar structures, it seemed likely that these properties might be related to their fold. In order to map the aggregation propensity and predicted disorder to the structure of the S100 proteins, the position specific scores were averaged and represented alongside with the secondary structure (Figure 2A). Position specific representation of averaged aggregation propensity (red bars) and averaged disorder (blue trace) show that high aggregation propensity regions (Z_agg_>1) are located in helices α1 and α4, contiguous to locally disordered segments (P>0.5). This is evident within helix α1 whose C-terminal side residues have high aggregation scores (residues in red boxes, Zagg1) but are flanked by disordered regions on both sides (residues in blue boxes, P>0.5). The other region with high aggregation propensity is located in helix α4 which is although being ordered is involved in target recognition interactions [16], thus being prone to structural variations. Although these regions are those common in the analyzed S100 protein, the fact is that additional amino acids stretched in some of these proteins such as the S100A9 C-terminal tail [39] may also play a role. Predictions of aggregation prone segments by Waltz [29] are coincident with these observations (data not shown). Interestingly, interactions between S100 proteins and their functional targets map to both ordered and disordered segments, the former mostly involved in target recognition and the latter in target contacts [16]. This puts in evidence the key role played by local structural disorder in S100 functions and structural mapping of these different regions illustrates how the structures of S100 proteins have evolved so as to accommodate in a stable globular fold sequences in contiguous positions with distinct physical and chemical propensities towards aggregation and disorder (Figure 2B). One interesting observation is that the segments with high aggregation propensity within helices α1 and α4 are located at the dimer interface, hence *a priori* protected by the quaternary structure from interactions that would promote β-aggregation (Figure 2C). Nevertheless, the high flexibility and exposure of nearby disordered segments is likely to result in structural rearrangements promoting loosening of this stabilization. Indeed, aggregation-prone segments have been suggested to be essential for protein folding and for mediating protein-protein interactions [40]. Herein lies what has been coined as the danger of structural disorder, as the increased dynamics and flexibility of disordered regions may, in some conditions, promote the formation of aggregation prone conformations [17,18].

### Acidic destabilization promotes S100 β-aggregation

**Figure 3:**
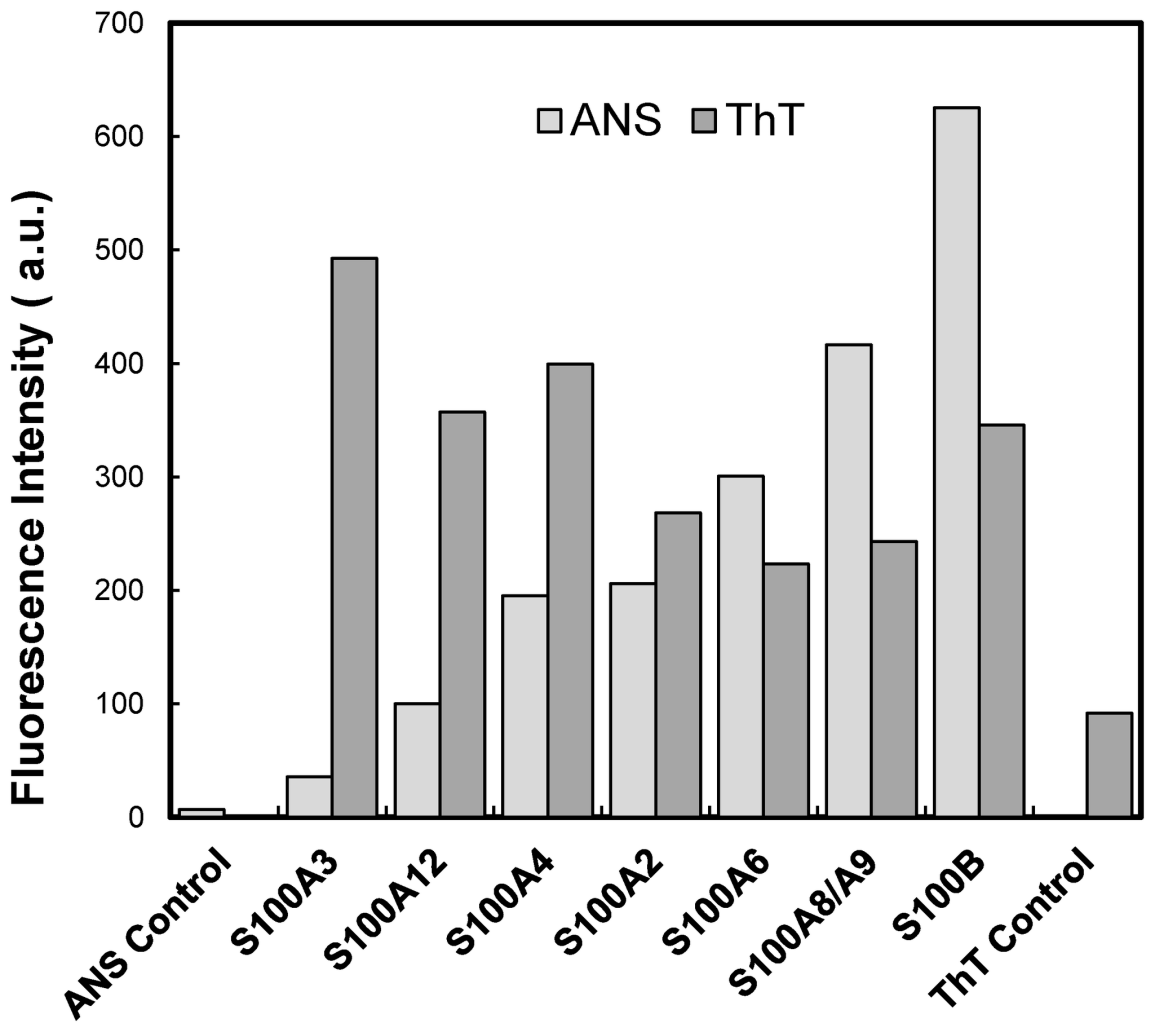
Fluorescence intensity of ThT and ANS bound to S100 proteins. The bars denote ThT (dark grey) and ANS (light grey) fluorescence upon diluting the different S100 proteins (1 mg/ml) in acidic buffer (glycine pH 2.5) at up to 30 min.

In order to study the formation of β-aggregates by S100 proteins we have destabilized the fold of the studied group of proteins through acidification, a well-established trigger for promoting aggregation. This has precisely been demonstrated for S100A6, which was shown to undergo amyloid β-aggregation both at neutral and acidic pH, albeit with very distinct timescales [23]. Indeed, S100A6 has been included in the present study as a control and was further analyzed in respect to structural changes occurring upon acidification. We have observed that lowering the pH has an impact on the protein quaternary structure, leading to dissociation of the dimer into soluble monomers, as determined by size exclusion chromatography (Figure S1). Such a formation of S100 monomers has been observed for S100B and S100A11 at low ionic strength, while keeping the α-helical fold [41]. The dimerization plane of S100 proteins is composed of highly conserved hydrophobic residues and we have noticed substantial solvent-accessible hydrophobic surfaces in the studied proteins under acidic conditions, as inferred from the emission increase of ANS, a molecule which becomes fluorescent upon interacting with structured hydrophobic moieties in proteins (Figure 3). Nevertheless, folding is retained: structural analysis of S100A6 by ATR-FTIR indicates that acidic conditions do not unfold the protein keeping the overall α-helical structure and evidence the formation of intermolecular β-sheets (Figure S2). This observation indicates that, as predicted by the computational and structural analysis, upon loss of protection from the quaternary structure (Figure 2C), the high aggregation prone segments within helices α1 and α4 may interconvert into β-aggregation prone conformers. In agreement, S100 proteins bind significantly the fluorophore ThT, a well-known reporter for the formation of β-aggregates which shows intensive fluorescence upon intercalation into stacked β-sheets that form during aggregation (Figure 3). Interestingly, a number of reports describe the use of ANS for amyloid fibril detection, as it binds to amyloid fibrillar or pre-fibrillar states [42] as well as to amyloid fibrils, being actually an effective *in vivo* sensor for β-aggregation [43]. Therefore, the intense ANS binding observed by the S100 proteins may reflect binding to presumable molten-aggregate states, as defined in [38]. Altogether, these results denote that amyloid competent aggregates are formed upon acidification, in agreement with the exposure of aggregation prone moieties that prompt β-sheet stacking.

### Acidic S100 β-aggregation is a fast kinetic process

**Figure 4:**
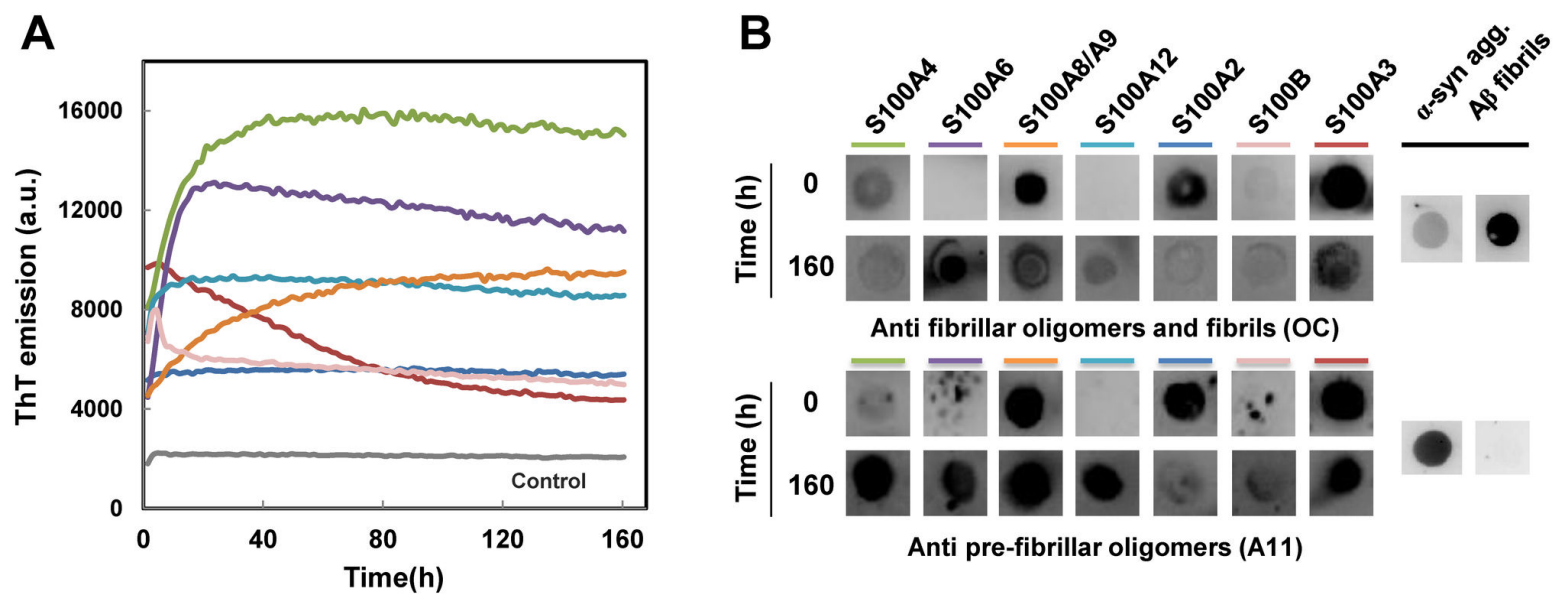
ThT binding kinetics and dot blot analysis of amyloid epitopes. A. S100 proteins (1 mg/mL) were incubated at pH 2.5 and 37°C during 160 h without agitation in the presence of ThT. The enhancement of ThT fluorescence at 480 nm was used to monitor amyloid formation, and the different proteins are color coded in the figure: S100A2 (dark blue), S100A3 (red), S100A4 (green), S100A6 (purple), S100A8/A9 (orange), S100A12 (light blue), S100B (pink). S100A12 is the protein undergoing faster ThT binding (*k*_app_= 0.38 h^-1^), S100A6 (*k*_app_= 0.14 h^-1^) and S100A4 (*k*_app_= 0.09 h^-1^) are slower, with comparable fibrillation rates, and finally S100A8/A9 has the slowest fibrillation rate (*k*_app_= 0.03 h^-1^). B. After incubation under amyloidogenic conditions, the conformation of S100 proteins was probed in a dot blot assay with the conformation-sensitive antibodies OC (anti fibrillar oligomers and fibrils) and A11 (anti pre-fibrillar oligomers). α-synuclein aggregates (oligomers and fibrils) and Aβ (fibrils) are included as controls.

We then analyzed the kinetics of S100 β-aggregation under acidic conditions, monitoring ThT binding as a function of time (Figure 4A). No lag phase was observed for any of the studied proteins, which reflects an instantaneous nucleation process and suggests that these proteins are able to form growth nuclei directly from their soluble conformations [32,38]. This is in full agreement with the model involving destabilization of S100 dimers. Different kinetic behaviors were observed: for one group of S100 proteins including S100A4, S100A6, S100A8/A9 and S100A12 ThT fluorescence increased exponentially as a function of time until a plateau phase was reached that then slowly decayed. In contrast a second group including S100A3, S100B and S100A2 showed a decay right at the start of the experiment. These kinetic traces are characterized by a slow time-dependent loss in ThT fluorescence, starting from a high level of initial binding. Such behavior could have multiple explanations. One possibility could be that aggregation excludes ThT binding sites at the aggregate core, resulting in its displacement, as proposed for the fibril formation of immunoglobulin light chains for which a similar kinetic behavior has been observed [44]. Another explanation could be related to dissociation phenomena of molecules from aggregates, which could become significant if the pool of starting building blocks becomes exhausted [32], a likely possibility considering the very fast initial rate. Finally, in the fibrillation reaction of glucagon, the development of fibrillar structures with increased ThT binding is followed by a decrease in ThT binding which the authors attribute to structural rearrangements of aggregates into different types of amyloid like fibrils with fewer ThT binding sites [45].

On the other hand, the ThT kinetics of the first group of S100s can be described by a mono exponential function for which different apparent rate constants (*k*_app_) were determined. S100A12 is the protein undergoing faster ThT binding (*k*_app_= 0.38 h^-1^), whereas S100A6 (*k*_app_= 0.14 h^-1^) and S100A4 (*k*_app_= 0.09 h^-1^) are slower, with comparable fibrillation rates, and finally S100A8/A9 has the slowest fibrillation rate (*k*_app_= 0.03 h^-1^). These results suggest that early aggregates formed upon acidification undergo further polymerization reactions that result in the buildup of increasingly ordered aggregates, as denoted by the increase in ThT binding reflecting intercalation into cross-β structures.

To further investigate the amyloid species formed we have carried out a dot blot analysis using conformation-specific antibodies which recognize generic structural epitopes specific to fibrils and fibrillar oligomers (OC antibody) or pre-fibril oligomers (A11 antibody) [34]. Pre-fibrillar oligomers are described as an assembly state that can result in fibrils upon subsequent concerted conformational conversion, while fibrillar oligomers will grow into fibrils through elongation by addition of monomeric blocks [34]. The data obtained show that acidification of S100 proteins results in the prompt formation of reactive epitopes towards both types of antibodies in S100A2, S100A3, S100A4 and S100A8/A9 (Figure 4B). These proteins thus form rather instantly (t= 0 h) amyloidogenic epitopes reactive towards OC and A11 antibodies, which are likely polydispersed mixtures of oligomers and fibers. None or residual initial reactivity for the two antibodies is observed for S100A6, S100A12 and S100B. The small scattered spots observed in S1004, S0100A6 and S100B for the A11 antibody at time zero were systematically observed in three independent experiments also when different membranes were used, suggesting that these may account for insoluble aggregates that do not deposit uniformly in the membrane when the samples are dotted. We observed that upon long term storage at pH 7, some S100 protein preparations develop aggregates for which we have observed reactivity against the OC and A11 antibodies. In this assay, after approximately seven days (t =160 h) all proteins developed into conformers reactive to both antibodies, although with distinct intensities. Strong reactivity to both OC and A11 antibodies is observed for S100A3, S100A6, S100A8/A9, whereas atypical weaker spots are noted for S100A2 and S100B.

### Structural diversity of S100 β-aggregates

**Figure 5:**
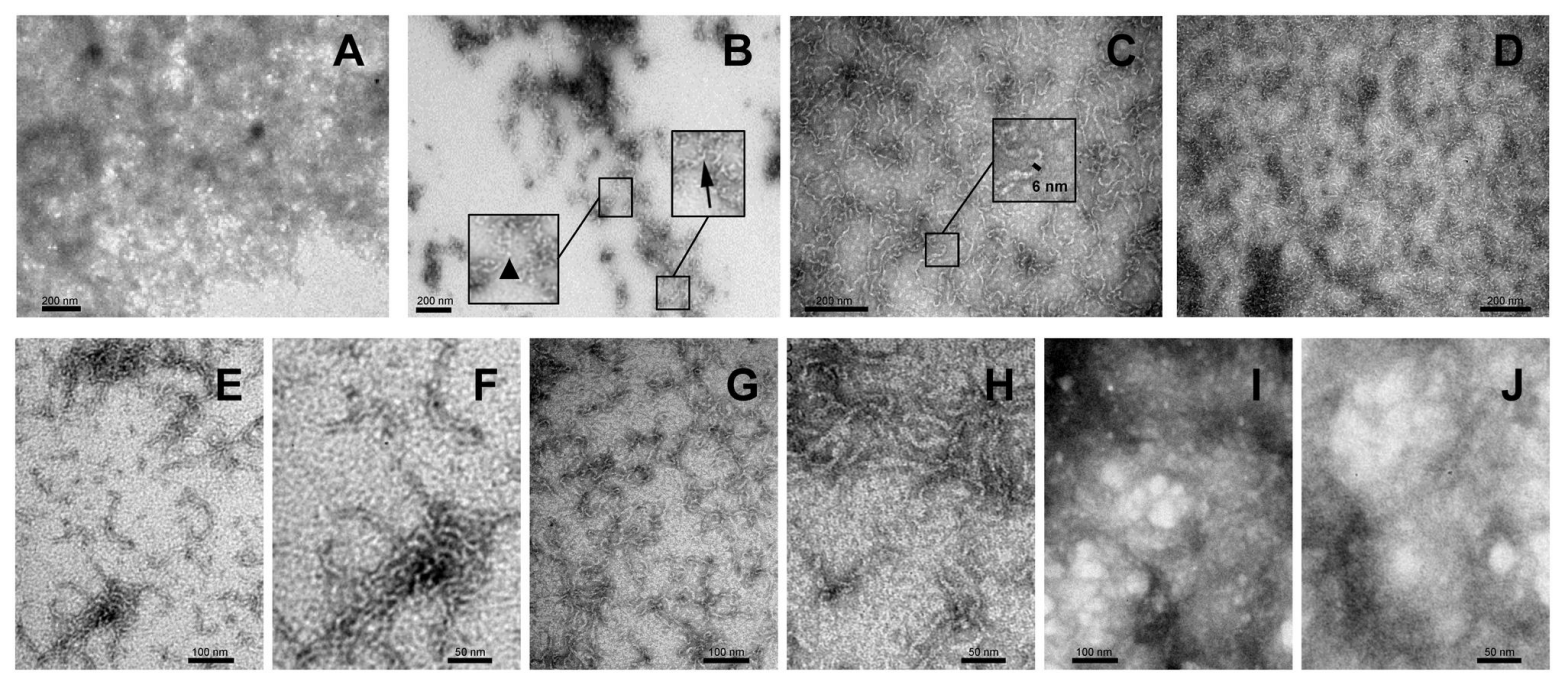
TEM imaging of S100 β-aggregates and amyloids. TEM images of S100 proteins (1 mg/mL) upon 160 h incubation at pH 2.5 and 37°C. (A) S100A2, (B) S100A3, (C) S100A4, (D) S100A6, (E) S100A8/A9, (F) S100A8/A9, magnification of panel E, (G) S100A12, (H) S100A12, magnification of panel G, (I) S100B and (J) S100B, magnification of panel I. Scale bars: 200 nm (panels A, B, C and D); 100 nm (panels E, G and I) and 50 nm (panels F, H and J). See text for details.

We then moved to characterize the properties of β-aggregates formed by S100 proteins at ultrastructural detail using transmission electron microscopy (TEM). In S100A2 preparations, we only observed large amorphous aggregates (Figure 5A) whereas S100A3 formed smaller aggregates, mixed with oligomers (Figure 5B, arrowhead); for S100A3 it was also possible to distinguish fibrillar-like structures 7-10 nm wide, emerging from the aggregates (Figure 5B, arrow). However, long protofibrils or fibrils were not detected for this incubation time. The remaining S100 proteins formed higher-ordered structures, in particular non-branched structures of fibrillar nature, although oligomeric species were also abundantly visualized. S100A4 formed the longest fibrils although a few oligomers were also easily visualized (Figure 5C); S100A6 produced apparent shorter fibrils but in significantly higher number than S100A4 (Figure 5D); S100A8/A9 (Fig. 5EF) and S100A12 (Fig 5GH) appeared as rich oligomeric preparations although there were fewer fibrils than in the S100A4 and S100A6 samples; S100A12 formed short fibrils although the species that protrude through the fibrils are aggregated forms (Fig. 5GH). Interestingly, S100A4, S100A6 and S100A8/9 exhibit both clear protofibril structure and enhanced ThT binding kinetics. Interestingly, these fibrillar structures appeared as curved species and although we could not unequivocally identify coiled fibrils. Nevertheless, and although we cannot rule out the hypothesis that these are protofibrils, the fact is that the diameter of the fibrillar structures depicted in Figure 5 panels C-H is 6-8 nm, which is characteristic of amyloid fibrils. Finally, S100B formed fibrils with bundles producing highly tangled and intertwined structures (Fig. 5IJ). These fibril structures likely formed very rapidly and gave rise to insoluble precipitates as evidenced by the ThT decay and dot blot analysis (Figure 4), also suggesting that S100B might undergo a different amyloidogenic pathway. Altogether, TEM and dot blot results revealed heterogeneous amyloidogenesis processes in S100 proteins, generating polymorphic oligomeric and fibrillar structures.

## Discussion

We here show that several human S100 proteins are able to undergo β-aggregation, a process which might occur *in vivo* as a cause or consequence of pathophysiological states. Our studies have been prompted by the recent proposal that, in spite of their globular fold, S100 proteins might contain regions of intrinsic disorder [16], alongside with the observations that a few S100 have the potential to oligomerize into amyloid assemblies [22]. The relationship between intrinsic disorder and aggregation into amyloids is well known for proteins forming insoluble protein deposits in neurodegenerative disorders, such as the Alzheimer β-peptide and α-synuclein [17,46-48]. We hypothesized that a similar mechanism could apply the S100 proteins. Indeed, the data collected so far indicate that β-aggregation of S100 proteins could arise from its dynamic fold and regions prone to intrinsic disorder. From the aggregation kinetics of S100 proteins one can infer that upon loss of structural protection an ensemble of amyloid competent conformations is formed by exposure of the otherwise solvent-inaccessible sequence segments with high aggregation propensity. These might have already some cross β-sheet organization, as denoted from prompt ThT binding. The high degree of dynamic fluctuations arising from the intrinsically disordered regions, and the high exposure of hydrophobic clusters which become exposed to the solvent, results in fast formation of a polydispersed mixture of oligomers and fibers. Loosening of the S100 dimer contacts, as evidenced by an increase in ANS fluorescence and shown by size exclusion chromatography for S100A6, is likely one of the triggers for S100 aggregation. Interestingly, aggregation pathways involving dissociation of oligomers are well-established for several proteins undergoing pathological β-aggregation, such as transthyretin [49,50] and SOD1 [51,52], respectively involved in familial amyloidotic polyneuropathy (FAP) and ALS, two amyloid-forming diseases. S100 proteins display variable expression levels which result in a range of physiological concentrations from pico to low micromolar thus overlapping the range of known dissociation constants (nano to micromolar) for the S100 dimers [1]. It is therefore likely that monomers occur *in vivo* which might be prone for aggregation. In this sense, S100 aggregation phenomena may carry important pathological implications. Amyloid oligomers are ubiquitous toxic species which can mediate multiple cytotoxic processes or trigger chronic inflammation as has been proposed for S100A8/A9 [53].

## Supporting Information

Figure S1 **Size exclusion chromatography of S100A6 at neutral and acidic pH.**
(TIFF)

Figure S2 **ATR-FTIR analysis of S100A6 upon acidification in the amide I region (pH 7.5, black; pH 2.5, red).**
(TIF)
