# Supplementary figures and images for: Intrinsically Disordered and Aggregation Prone Regions Underlie β-Aggregation in S100 Proteins
Source: PLoS One. 2013 Oct 1;8(10):e76629. doi: 10.1371/journal.pone.0076629 (PMC3788126; doi:10.1371/journal.pone.0076629)

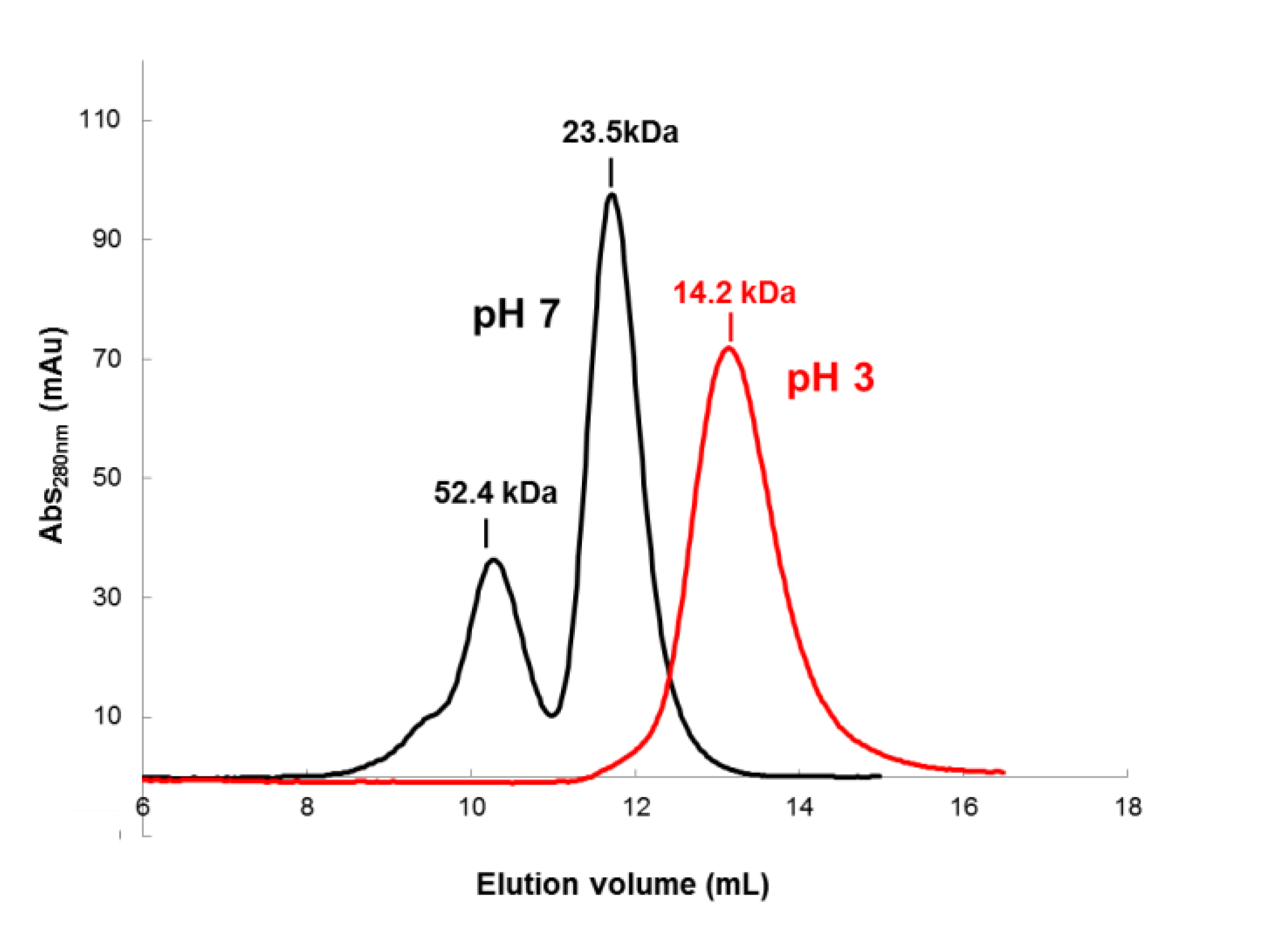

Supplement: Figure S1 — Size exclusion chromatography of S100A6 at neutral and acidic pH. (TIFF) [file pone.0076629.s001.tiff]

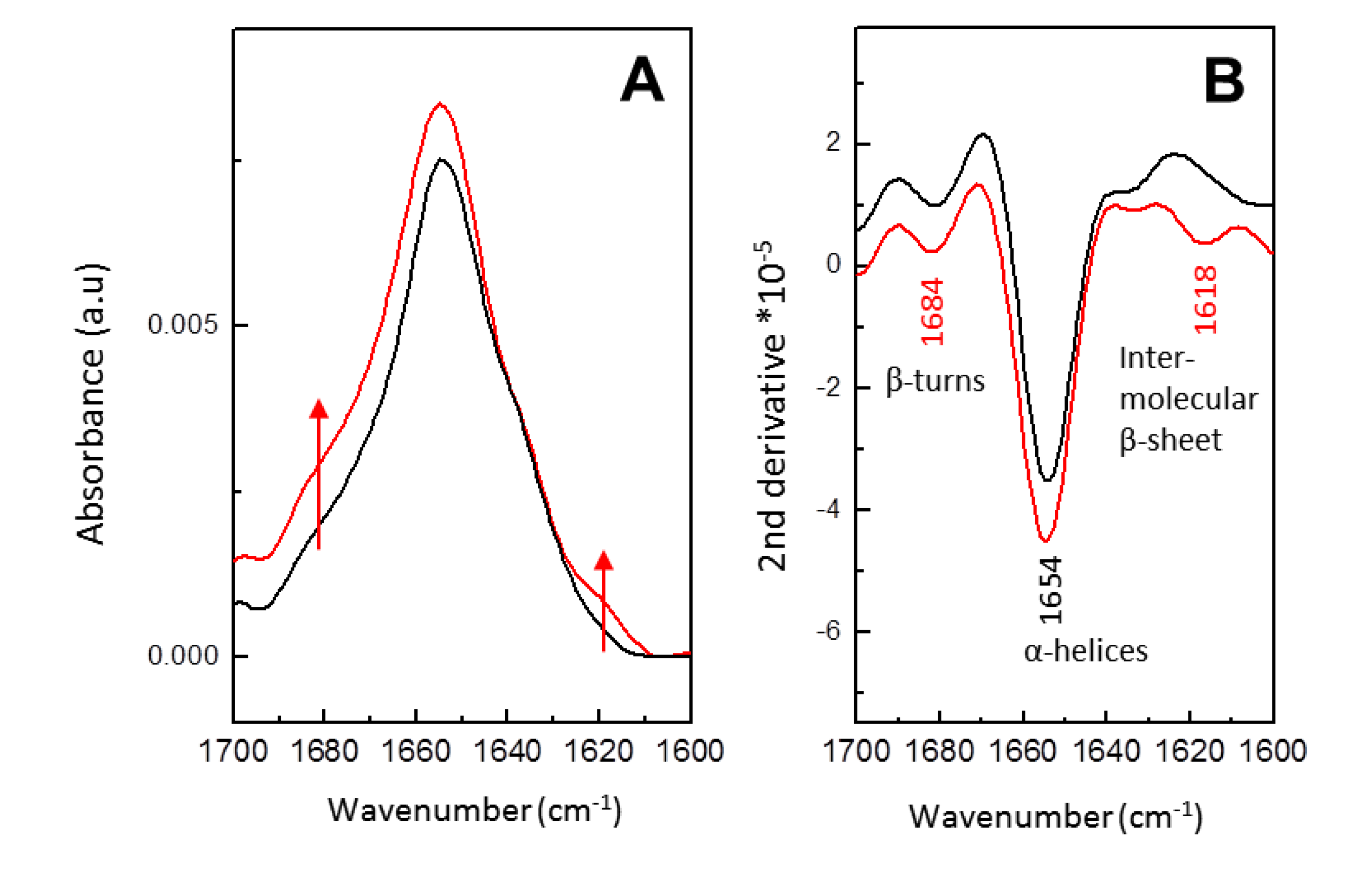

Supplement: Figure S2 — ATR-FTIR analysis of S100A6 upon acidification in the amide I region (pH 7.5, black; pH 2.5, red). (TIF) [file pone.0076629.s002.tif]
